# Mechanical Conditioning (MeCo) Score Progressively Increases Through the Metastatic Cascade in Breast Cancer via Circulating Tumor Cells

**DOI:** 10.3390/cancers17101632

**Published:** 2025-05-12

**Authors:** Ghassan Mouneimne, Casey Connors, Adam Watson, Adam Grant, Daniel Campo, Alexander Ring, Pushpinder Kaur, Julie E. Lang

**Affiliations:** 1 University of Arizona Cancer Center, University of Arizona, Tucson, AZ 85724, USA; 2 Division of Breast Surgery, Cleveland Clinic, Lerner College of Medicine, 9500 Euclid Ave., Cleveland, OH 44195, USA; 3 MeCo Diagnostics, San Diego, CA 92103, USA; adam@mecodiagnostics.com (A.W.);; 4 Norris Comprehensive Cancer Center, University of Southern California, 1441 Eastlake Ave., Los Angeles, CA 90033, USApushpinderkaur2006@gmail.com (P.K.); 5 Department of Medical Oncology and Hematology, University Hospital Zürich, Rämistrasse 100, 8091 Zurich, Switzerland

**Keywords:** breast cancer, circulating tumor cell, CTC, mechanical conditioning score, MeCo score, biomarker

## Abstract

Background: The mechanical conditioning (MeCo) score is a multigene expression signature that is acquired by cancer cells in the primary breast tumor and is reflective of their responsiveness to ECM stiffness caused by tumor fibrosis. Chromatin remodeling downstream of mechanotransduction allows cancer cells to retain these acquired aggressive features even in the absence of mechanical stimulation from the primary tumor microenvironment, for instance, after dissemination through systemic circulation during metastasis. Importantly, patients who have high MeCo score tumors are at higher risk of developing metastatic breast cancer, compared to those with low MeCo scores. Moreover, circulating tumor cells (CTCs) are associated with a higher rate of metastatic dissemination, making CTC detection in the circulation of patients with breast cancer a significant prognostic biomarker for breast cancer metastasis. Beyond their enumeration per blood volume units, specific prognostic features of CTCs are not fully explored. We sought to determine whether MeCo scores increase stepwise along the metastatic cascade, from primary tumors to CTCs to distant metastatic colonization, using patient-matched biopsies. Methods: CTCs were isolated from the peripheral blood of two patient cohorts: patients with early-stage breast cancer using immunomagnetic enrichment/FACS methodology; and patients with late-stage breast cancer using the ANGLE Parsortix microfluidics system. Gene expression profiling using RNA-seq was performed on CTCs and matched primary tumors (PTs) in the early-stage cohort, and on CTCs and matched metastases (METs) for the late-stage cohorts. A quantile normalization approach was used to allow comparison across cohorts and MeCo scores were computed for all samples. The Wilcoxon matched-pairs signed rank test was performed for the comparison of MeCo scores from matching samples within each cohort; the Mann–Whitney unpaired test was used to compare MeCo scores of CTCs across cohorts. Results: In 12 pairs of patients with early-stage breast cancer, MeCo scores in CTCs were significantly higher than in their matched PTs (*p* = 0.026). Additionally, in 26 pairs of metastatic patient CTCs and METs, MeCo scores were significantly higher in METs compared to matched CTCs (*p* = 0.0004). MeCo scores of CTCs were similar between patients with early- and late-stage breast cancers, despite differing CTC isolation strategies (epitope-dependent and microfluidics size gradient). Notably, 98% of the genes in the MeCo score were present across evaluable CTC, MET, and PT samples. Conclusions: Our results show that the MeCo score is higher in CTCs than in PTs, and higher in METs compared to CTCs, in early- and late-stage breast cancer, respectively (i.e., PT < CTC < MET). Therefore, the MeCo score is progressively higher throughout the metastatic cascade in breast cancer. These findings demonstrate that mechanical conditioning from primary tumors is retained during metastatic progression, after mechanical induction by ECM stiffness is lost, as cancer cells disseminate through systemic circulation. Additionally, these findings support that cancer cells with higher MeCo scores are more competent with—and potentially selected for—metastatic progression. Importantly, these findings provide a novel feature of CTCs, mechanical conditioning (MeCo), which is associated with higher capacity for metastasis. Furthermore, since the CTC MeCo score is elevated even in early-stage breast cancer, it could provide, in addition to CTC enumeration, a potential prognostic indicator to improve metastatic risk assessment in early disease.

## Simple Summary

Although circulating tumor cell (CTC) enumeration via liquid biopsy has been shown to serve as a prognostic and predictive biomarker, insights into CTC biology have been scarce. The mechanical conditioning (MeCo) score is a multigene expression signature reflective of the cancer cell response to fibrotic extracellular matrix (ECM) stiffness and is associated with breast cancer survival. We evaluated two available datasets from early and metastatic breast cancer with whole transcriptome RNA-Seq data from CTCs for the quantification of the MeCo score. The MeCo score was higher in CTCs compared to matched primary tumors in stage II–III patients, and even higher in metastatic biopsies compared to matched CTCs in stage IV patients, revealing a stepwise increase along the metastatic cascade. These findings support the notation that cancer cells with higher MeCo scores are more competent for—and potentially selected during—metastatic progression.

## 1. Introduction

In addition to primary breast tumor’s intrinsic biologic features, the surrounding tumor microenvironment plays a crucial role in influencing aggressive behavior, tumor progression, and eventual metastatic spread [1,2,3]. The tumor microenvironment comprises a myriad of elements, including extracellular matrix (ECM), immune cells, fibroblasts, and blood vessels [1,2,4,5]. When mechanical changes in the ECM occur due to tumor fibrosis, genetic and epigenetic alterations occur within cancer cells, promoting their growth and invasion [1,6,7,8]. This process of biomechanical changes driving gene expression programs prompted the development of the mechanical conditioning score (MeCo score), first described by Watson et al., which comprises 1004 genes that are both stiffness- and metastasis-associated [6]. The MeCo score reflects the gene expression profile acquired in response to a stiff ECM, with high MeCo scores maintained via stable chromatin remodeling, even after cancer cell dissemination away from the inciting ECM [6]. As previously illustrated by Watson et al., patients with breast cancer having high MeCo scores are at a greater risk of bone metastasis (*p* < 0.0001; HR = 2.2, 95% CI 1.7–2.7) [6].

The study of CTCs through liquid biopsy has revealed their value as prognostic and predictive biomarkers, though their biological properties remain incompletely characterized [9]. CTCs can be evaluated using a variety of downstream techniques to characterize their biology through studies of RNA, DNA, or protein [10]. HER2 expression in CTCs has been shown to be a noninvasive marker of bone metastasis [11]. Gene expression profiling of CTCs is of interest since (1) not all DNA mutations are expressed, and (2) it provides a better understanding of the tumor biology of cancer cells present in peripheral blood. Our team has previously reported the feasibility of gene expression profiling of CTCs with classification of their intrinsic subtype using the PAM50 and Risk of Recurrence (ROR) [12]. Relatively few studies have performed whole-transcriptome RNA-Seq of CTCs in breast cancer [13,14]. As CTCs are the precursors of metastasis, some groups have identified metastatic signatures in CTCs [15], with the goal of identifying CTC-directed therapeutics in the future.

Our team have previously published two studies demonstrating successful whole-transcriptome RNA-Seq analysis of CTCs in both early-stage and metastatic breast cancer [13,14]. However, the mechanical conditioning properties of CTCs, as measured using the MeCo score, have not been explored. This information may provide insight into the metastatic risk of patients with early-stage breast cancer and could serve as a predictive biomarker of aggressive biology. The present study sought to determine whether the high MeCo scores observed in primary tumors are retained in the CTCs of patients with early-stage breast cancer and to compare MeCo scores in CTCs versus metastatic biopsy samples from patients with late-stage breast cancer.

## 2. Methods and Materials

### 2.1. Patient Cohorts and Sample Collection

CTCs were isolated from the peripheral blood of two patient cohorts. The first cohort included patients with stage II–III breast cancer [13,14]. The second cohort was comprised of patients with metastatic breast cancer [13]. These cohorts were selected since they both utilized a rapid processing strategy in which peripheral blood specimens collected in EDTA tubes were processed immediately for CTC isolation to avoid RNA degradation. The total time from blood draw to CTC harvest did not exceed three hours in these two cohorts. Patient samples were collected at baseline prior to treatment in both cohorts. Samples were collected after IRB approval at the University of Southern California under a blood biorepository protocol (IRB HS-14-00595 and HS-11-00208). Detailed clinical annotations for these cohorts with patient and tumor characteristics have been published previously [13,14]. A flowchart of sample collection and processing is shown in Supplementary Figure S1.

### 2.2. CTC Isolation Techniques

For the early-stage breast cancer cohort, CTCs were isolated using an immunomagnetic enrichment/FACS methodology, as previously described [13,16], using FACS reagents by BD Biosciences (San Jose, CA, USA). Samples underwent positive selection using EpCAM (MJ37) monoclonal antibodies conjugated to magnetic beads (ferrofluid). CTC selection criteria by FACS were based on strict gating using positive and negative controls thresholded for each sample run. Marker selection was performed gating with EpCAM (EBA1-PE)+/thioflavin t buffer dye+/CD45(2D1-PerCp Cy5.5)-phenotype. Cells were sorted directly into 5 μL of Prelude Direct lysis buffer (NuGEN Technologies, Inc., San Carlos, CA, USA) and stored at −80 °C for further use.

For the metastatic breast cancer cohort, CTCs were isolated using the ANGLE Parsortix microfluidics system [6]. Peripheral blood samples were processed through the Parsortix device, which captures CTCs based on size and deformability as they pass through a microsieve cassette with a critical gap of 10 μm. After isolation, cells were harvested from the cassette, and cell pellets were resuspended in 10 μL of Prelude Direct lysis buffer and stored at −80 °C for further use.

### 2.3. RNA Sequencing and Gene Expression Analysis

Gene expression profiling using RNA sequencing was performed on CTCs and on matched primary tumors (PTs) and metastases (METs) for the early-stage and late-stage cohorts, respectively. For the early-stage cohort, duplicate aliquots of 1 μL cell lysate were used as direct input for sequencing library preparation using the Ovation Single Cell RNA-Seq System (NuGEN). For peripheral blood, total RNA isolation was performed using the QIAamp RNA Blood Mini Kit (QIAGEN, Hilden, Germany). RNA-seq library preparation for peripheral blood and PTs was performed using the NuGEN Ovation RNA System V2 and the NuGEN Ultra Low Library System V2. The quality and quantity of the amplified libraries were evaluated using a Qubit fluorometer (Invitrogen, Carlsbad, CA, USA) and an Agilent Bioanalyzer 2100 (Agilent Technologies, Santa Clara, CA, USA). For the metastatic cohort, sequencing libraries were prepared using either 50 ng of RNA extracted from a metastasis or peripheral blood, isolated with the TRIzol or RiboPure kits (both from Thermo Fischer Scientific, Waltham, MA, USA), respectively, or 2 μL of CTC lysate. Library preparation was performed using the Ovation RNA-Seq System V2 and the Ovation Ultralow Library System V2 (NuGEN).

All libraries were sequenced using an Illumina HiSeq 2500 at the University of California, Los Angeles, Clinical Microarray Core, with 100 bp paired-end reads. FASTQ files were stored on the High-Performance Computing Cluster of the USC.

### 2.4. PAM50 Subtyping and MeCo Score Calculation

PAM50 intrinsic subtyping and risk of recurrence (ROR) were previously reported [17,18] and are used here for comparison with the MeCo score. In our early-stage cohort, primary tumor PAM50 subtyping was performed with NanoString assays (NanoString Technologies, Seattle, WA, USA [17]). Breast cancer molecular subtype classification was predicted using the open-source genefu package in R/Bioconductor (www.bioconductor.org; 2018 and 2022) for both the NanoString (from formalin-fixed, paraffin-embedded primary tumor samples) and RNA-seq assays (from CTCs [18] and metastatic biopsies in our early-stage and metastatic cohorts). Data were downloaded as FASTQ files from the Gene Expression Omnibus (GSE113890 and GSE111842) to query for MeCo score genes (*n* = 1004 genes) [6]. Paired *t*-tests were used to compare normalized (z-score) MeCo scores computed from RNA-seq data between paired CTC and PT biopsies. Patients were ranked based on their MeCo-CTC scores and then split at the median into two clusters: Low MeCo-CTC cluster and High MeCo-CTC cluster. Two-way ANOVA and Fisher’s LSD test for multiple comparisons were used. A paired *t*-test was also used to compare normalized (z-score) MeCo scores computed using RNA-seq data from paired CTC and MET biopsies. Patients were ranked based on MeCo-CTC scores and were also split at the median into two clusters: Low MeCo-CTC scores and High MeCo-CTC scores. Two-way ANOVA and Fisher’s LSD test were used for multiple comparisons.

To address potential batch effects and technical variations between the two isolation methods, a quantile normalization approach was used to minimize methodological bias while preserving biological differences. This approach transforms the distributions of gene expression values across samples to follow the same distribution, effectively minimizing technical variation while maintaining relative gene expression relationships within each sample. We further validated our approach by confirming that 98% of the genes in the MeCo signature were detectable across all sample types, regardless of the isolation method. Additionally, we performed principal component analysis on the normalized data to verify that sample clustering was driven by biological factors rather than technical differences between isolation platforms. While we acknowledge the limitations of cross-platform comparisons, the consistent biological patterns observed across both cohorts (with MeCo scores progressively increasing from PT to CTC to MET) suggest that our findings represent true biological phenomena rather than technical artifacts.

In addition, MeCo scores were computed and normalized for all patients in the METABRIC dataset [19], and analysis was performed by comparing MeCo scores across stage and grade [19].

## 3. Results

### 3.1. MeCo Score Comparison Between Primary Tumors and CTCs in Early-Stage Breast Cancer

**Figure 1:**
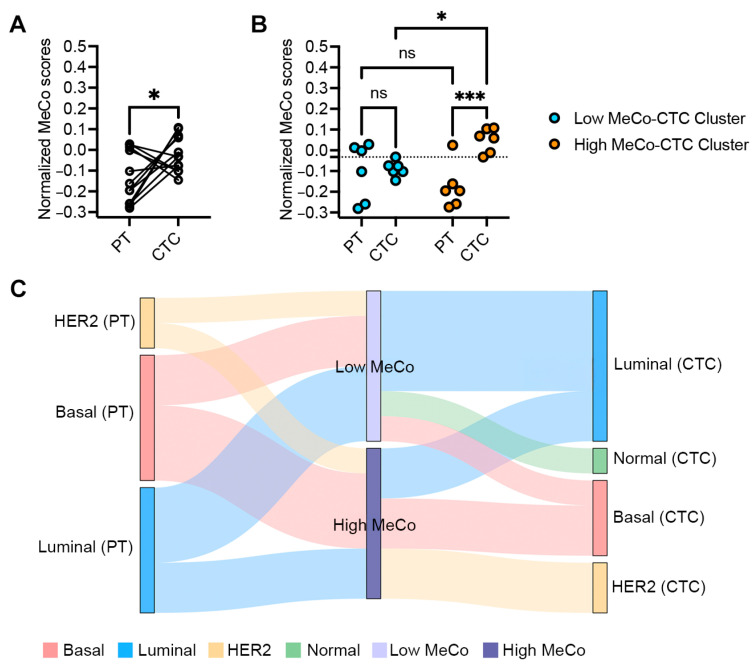
(**A**): Assessing difference in MeCo scores between CTCs and PTs in stage II–III breast cancer. A paired *t*-test was used to compare normalized (z-score) MeCo scores computed from RNA-seq data of paired CTC and PT biopsies. MeCo scores in CTCs (MeCo-CTC scores) were higher than those in paired PTs from the same patients; * *p* = 0.028, using a paired *t*-test. (**B**): Assessing differences in MeCo scores in Low and High MeCo-CTC patient clusters. Patients were ranked based on MeCo-CTC scores and then split at the *median* into two clusters: Low MeCo-CTC cluster and High MeCo-CTC cluster. The dotted line indicates the median MeCo-CTC score. Two-way ANOVA and Fisher’s LSD test for multiple comparisons were used; * *p* = 0.020 and *** *p* = 0.0005. (**C**): Transitions between PAM50 subtypes and MeCo score categories in paired primary tumor-CTC samples. This Sankey diagram illustrates the transitions between PAM50 molecular subtypes and MeCo score categories in matched primary tumor (PT) and circulating tumor cell (CTC) samples from 12 patients with early-stage breast cancer. The width of each flow is proportional to the number of patients in each category. Left nodes represent PAM50 subtypes in primary tumors (basal, luminal, and HER2). Middle nodes categorize MeCo scores as either high or low, divided at the median MeCo score value (−0.179). Right nodes represent PAM50 subtypes in matched CTCs (basal, luminal, HER2, and normal).

Analysis of twelve matched pairs of stage II–III breast cancer samples revealed significantly higher MeCo scores in CTCs compared to their corresponding primary tumors (PTs) (*p* = 0.028) (Figure 1A). Individual patient data showed considerable heterogeneity, with PT scores ranging from −0.28 to 0.03 and CTC scores from −0.15 to 0.11. Upon stratification by the median MeCo-CTC scores, patients in the High MeCo-CTC cluster demonstrated significantly higher scores compared to their matched PTs, while this difference was not significant in the Low MeCo-CTC cluster (Figure 1B). PAM50 molecular subtype analysis revealed molecular heterogeneity, with 58% of cases (7/12) showing discordant subtypes between PT and CTC samples (Figure 1C, Table S1).

### 3.2. MeCo Score Comparison Between CTCs and Metastatic Sites in Stage IV Breast Cancer

**Figure 2:**
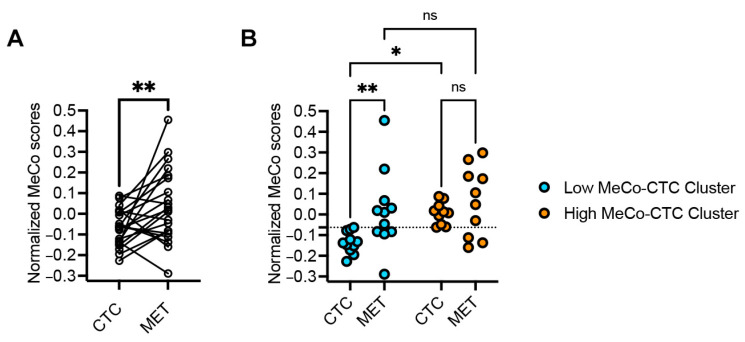
(**A**): Assessing the difference in MeCo scores between CTCs and METs in stage IV breast cancer. A paired *t*-test was used to compare normalized (z-score) MeCo scores computed using RNA-seq data from paired CTC and MET biopsies. ** *p* = 0.0068. **(B**): Assessing the difference in MeCo scores of METs vs. CTCs in *Low* and *High* MeCo-CTC patient clusters. Patients were ranked based on MeCo-CTC scores and then split at the median into two clusters: Low MeCo-CTC scores and High MeCo-CTC scores. The dotted line shows the median MeCo-CTC score. A two-way ANOVA and Fisher’s LSD test for multiple comparisons were used; * *p* = 0.019 and ** *p* = 0.0096.

In the metastatic cohort (26 paired samples), MeCo scores of metastatic sites (METs) were significantly higher than those of their matching CTCs (*p* = 0.0068) (Figure 2A). This pattern was most pronounced in the Low MeCo-CTC cluster, where MET scores were significantly elevated compared to paired CTCs (*p* = 0.0096). Notably, patients in the High MeCo-CTC cluster showed no significant difference between MET and CTC scores, suggesting a potential plateau effect in mechanical conditioning during metastatic progression (Figure 2B). This pattern remained consistent despite molecular subtype discordance between CTC and MET samples in 65% of cases, highlighting the robustness of mechanical conditioning as a feature of metastatic progression. We found no conclusive correlation between MeCo scores and ROR.

### 3.3. Correlation Between MeCo Scores and Established Prognostic Factors

Notably, MeCo scores in CTCs showed consistency across disease stages despite the use of different isolation platforms (immunomagnetic enrichment/FACS for early stage versus ANGLE Parsortix for metastatic patients). Technical validation through RNA-seq analysis demonstrated robust detection of MeCo signature genes, with 98% coverage across all sample types (CTC, MET, and PT), supporting the reliability of MeCo score assessment regardless of the isolation method.

**Figure 3:**
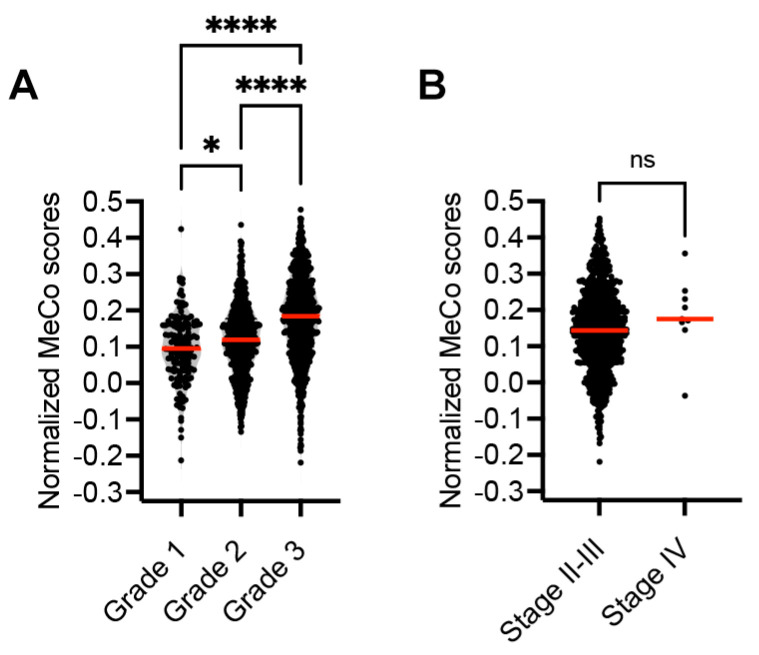
(**A**): Assessing MeCo score distribution across tumor grades in the METABRIC dataset. (**A**): MeCo scores increase with tumor grade (G1–G3). Grade 1 tumors (*n* = 154) showed a mean score of 0.094 ± 0.092, Grade 2 (*n* = 660) 0.117 ± 0.096, and Grade 3 (*n* = 821) 0.179 ± 0.116. (**B**): Assessing MeCo score distribution across tumor stage in the METABRIC dataset. Comparison between non-metastatic (Stages II–III, *n* = 785, mean = 0.146 ± 0.114) and metastatic disease (Stage IV, *n* = 9, mean = 0.185 ± 0.105). Box plots show the median, quartiles, and range; points indicate individual values. * *p* < 0.05 and **** *p* < 0.0001.

Analysis of the METABRIC dataset revealed a significant association between tumor grade and MeCo scores, with higher-grade tumors displaying elevated scores (Figure 3). While there was a trend toward higher MeCo scores in stage IV disease compared to stages II–III, this difference did not reach statistical significance, possibly due to the limited number of stage IV samples (*n* = 9) in the METABRIC cohort or because mechanical conditioning reflects intrinsic tumor biology rather than anatomical disease extent.

Collectively, these results demonstrate a progressive increase in MeCo scores throughout the metastatic cascade, from primary tumors to CTCs to metastatic sites. This pattern persists across different molecular subtypes and isolation methods, suggesting that mechanical conditioning may be a fundamental feature of metastatic progression in breast cancer.

## 4. Discussion

In recent years, an emphasis on tumor biology has helped reshape treatment strategies in the management of breast cancer [20]. CTCs allow for evaluating tumor biology via a minimally invasive blood draw [21].

Watson et al. previously demonstrated that mechanical conditioning significantly influences breast cancer cell invasion, with cells exposed to different ECM stiffness showing distinct invasive behavioral patterns [1,6]. Mechanical conditioning is quantified using the MeCo score. In patients, high MeCo scores correlate with aggressive behavior, increased local invasion, and elevated risk of metastasis. Recent clinical evidence further supports the importance of mechanical conditioning in breast cancer progression. Quintela-Fandino et al. demonstrated that patients having HER2-negative breast cancer with high MeCo scores had significantly worse outcomes and higher relapse risk compared to low MeCo patients (HR = 0.21, *p* = 0.0075). However, neoadjuvant treatment with the anti-fibrotic drug nintedanib reduced MeCo scores by 25% and improved survival outcomes specifically in high MeCo patients, establishing the MeCo score as a potential predictive biomarker for anti-fibrotic therapeutic intervention [22].

Our study extends previous MeCo score analyses beyond primary tumors by examining CTCs and METs, providing new insights into the dynamics of mechanical conditioning during metastatic progression. Beyond their enumeration per blood volume units, specific prognostic features of CTCs have not been fully explored [23]. CTCs are associated with a higher rate of metastatic dissemination, making CTC detection in the circulation of breast cancer patients a significant prognostic biomarker for breast cancer metastasis [24]. An alternative to CTCs, circulating cell-free tumor DNA (ctDNA) is more commonly analyzed in patients with metastatic breast cancer through liquid biopsy. Liquid biopsy, which is collected through a blood draw, is used in patients with breast cancer to assess DNA mutations in genes such as PIK3CA and ESR1, which have FDA-approved targeted therapeutic interventions [24,25]. In non-metastatic patients, ctDNA tumor fraction has been shown to be prognostic of worse outcomes [26]. When analyzing shed ctDNA, there is no insight into gene expression, but rather only the presence of tumor-specific mutations, which is not directly reflective of dynamic tumor gene expression [27]. In fact, only a very small percentage of DNA mutations are expressed, which is why circulating tumor DNA and RNA from CTCs are not parallel assays [24]. Although there is great enthusiasm in the research community for liquid biopsy assays, there is no evidence that such testing improves survival outcomes for patients [28]. thus, studies testing the potential utility of liquid biopsy as a predictive biomarker (predicting benefit from specific therapies) are needed. The MeCo score is a predictive biomarker associated with treatment benefit from the anti-fibrotic drug nintedanib.

Our comprehensive analysis of MeCo scores across the metastatic cascade reveals a striking pattern of progressive increase from primary tumors through CTCs to metastatic sites (PT < CTC < MET), revealing that mechanical conditioning persists and intensifies even when cells are no longer exposed to the stiff matrix of the primary tumor. This pattern suggests that mechanical conditioning may serve as a selective pressure during metastatic progression, potentially identifying cells with enhanced metastatic capability. The persistence of elevated MeCo scores in CTCs, even in early-stage disease, suggests that mechanical conditioning is an early event in metastatic progression that is hardwired through epigenetic mechanisms, enabling cells to maintain their aggressive phenotype throughout the metastatic cascade.

Subtype shifts frequently occur between primary and metastatic tissues; therefore, it is not surprising that CTCs would also demonstrate changes in subtype. The discrepancies between PAM50 subtypes in matched primary tumor and CTC pairs reflect tumor heterogeneity and cellular plasticity during metastasis. While primary tumors may harbor minor subclones with enhanced intravasation capabilities that become overrepresented in CTCs, notably, MeCo scores remain elevated in CTCs despite these molecular subtype shifts, suggesting that mechanical conditioning represents a more stable feature than PAM50 classification. By combining CTC detection with MeCo score assessment, we may better identify patients at higher risk for metastatic progression, potentially enabling more precisely targeted therapeutic interventions.

Our analysis of the metastatic cohort revealed further increases in MeCo scores at metastatic sites compared to paired CTCs, suggesting continued selection for mechanical conditioning during metastatic colonization. Intriguingly, this increase was most pronounced in cases with Low MeCo-CTC scores, while High MeCo-CTC cases showed similar scores between CTCs and METs. This pattern suggests a potential ceiling effect in mechanical conditioning, where highly adapted cells may have already achieved optimal mechanical properties for metastatic progression.

These observations suggest a threshold effect in mechanical conditioning, where CTCs with initially higher MeCo scores may have already achieved optimal mechanical properties for metastatic colonization. Conversely, cells with lower MeCo scores appear to undergo further selection and adaptation during metastatic progression. The convergence of MeCo scores in metastatic sites, regardless of initial CTC scores, supports the hypothesis that successful metastatic colonization requires a certain threshold of mechanical conditioning.

Analysis of the METABRIC dataset provided additional insights into the relationship between mechanical conditioning and traditional prognostic factors. The strong correlation between MeCo scores and tumor grade, coupled with the lack of association with disease stage, suggests that mechanical conditioning reflects intrinsic tumor biology rather than anatomical progression. This observation aligns with emerging evidence that metastatic potential may be established early in tumor evolution, although we acknowledge the limited representation of stage IV samples in the METABRIC cohort.

Recent studies have further illuminated the complex relationship between mechanical properties and cancer metastasis [1]. The mechanical adaptability of cancer cells appears to be closely tied to their invasive potential [29]. The importance of ECM composition in regulating these mechanical interactions is highlighted by evidence that specific matrix components can significantly influence tumor progression and patient outcomes through their effects on the mechanical microenvironment [30]. These mechanical interactions appear to be particularly important during the early stages of metastatic progression, potentially influencing which cells are capable of successful dissemination [6,8].

The ability to assess mechanical conditioning through CTC analysis offers a potential new tool for predicting metastatic risk, particularly for bone metastasis [31]. Our results suggest that MeCo-CTC analysis could be especially valuable in early-stage disease, where identifying high-risk patients with additional insights could inform and improve treatment decisions. Furthermore, the relationship between mechanical conditioning and metastatic progression suggests that targeting ECM stiffness responses could represent a novel therapeutic strategy, particularly in tumors showing evidence of elevated mechanical conditioning.

## 5. Conclusions

Our study reveals mechanical conditioning as a dynamic feature that intensifies throughout breast cancer progression, with MeCo scores increasing from primary tumors to CTCs to metastatic colonization sites. By incorporating MeCo score analysis into CTC evaluation, we have identified a novel biomarker that may enhance our ability to predict metastatic potential. These findings not only provide new insights into the biology of metastatic progression but also suggest potential therapeutic strategies targeting mechanical conditioning using anti-fibrotics. Further investigation of MeCo-CTC scores in prospective clinical trials will help validate their utility in risk stratification and treatment strategy.

## Data Availability

Data were downloaded as FASTQ files from the Gene Expression Omnibus GSE113890 and GSE111842.

## Supplementary Materials

The following supporting information can be downloaded at: https://www.mdpi.com/article/10.3390/cancers17101632/s1, Supplementary Figure S1. Patient Cohort Flowchart. Schematic representation of sample collection and processing workflow for the two patient cohorts in this study. The early-stage cohort (left, blue) consisted of 12 paired primary tumor (PT) and circulating tumor cell (CTC) samples from stage II-III breast cancer patients. CTCs were isolated using immunomagnetic enrichment followed by FACS. The metastatic cohort (right, pink) consisted of 26 paired CTC and metastatic site (MET) samples from stage IV breast cancer patients. CTCs in this cohort were isolated using the ANGLE Parsortix microfluidics system. All samples underwent RNA extraction and sequencing, followed by MeCo score analysis to assess mechanical conditioning throughout the metastatic cascade. Table S1. Raw data table showing the corresponding PAM50 subtypes in CTCs and PTs, and the ROR in a cohort of patients with early-stage breast cancer. Table S2. Raw data table showing the corresponding PAM50 subtypes in a cohort of patients with metastatic breast cancer. A number of patients had MeCo scores and PAM50 subtype assessed at follow-up and from second metastases.
