# Supplementary material for: Mechanical Conditioning (MeCo) Score Progressively Increases Through the Metastatic Cascade in Breast Cancer via Circulating Tumor Cells
Source: Cancers (Basel). 2025 May 12;17(10):1632. doi: 10.3390/cancers17101632 (PMC12109637; doi:10.3390/cancers17101632)
Supplement: Supplementary file 1 [file cancers-17-01632-s001.zip › cancers-3487037-supplementary.pdf]

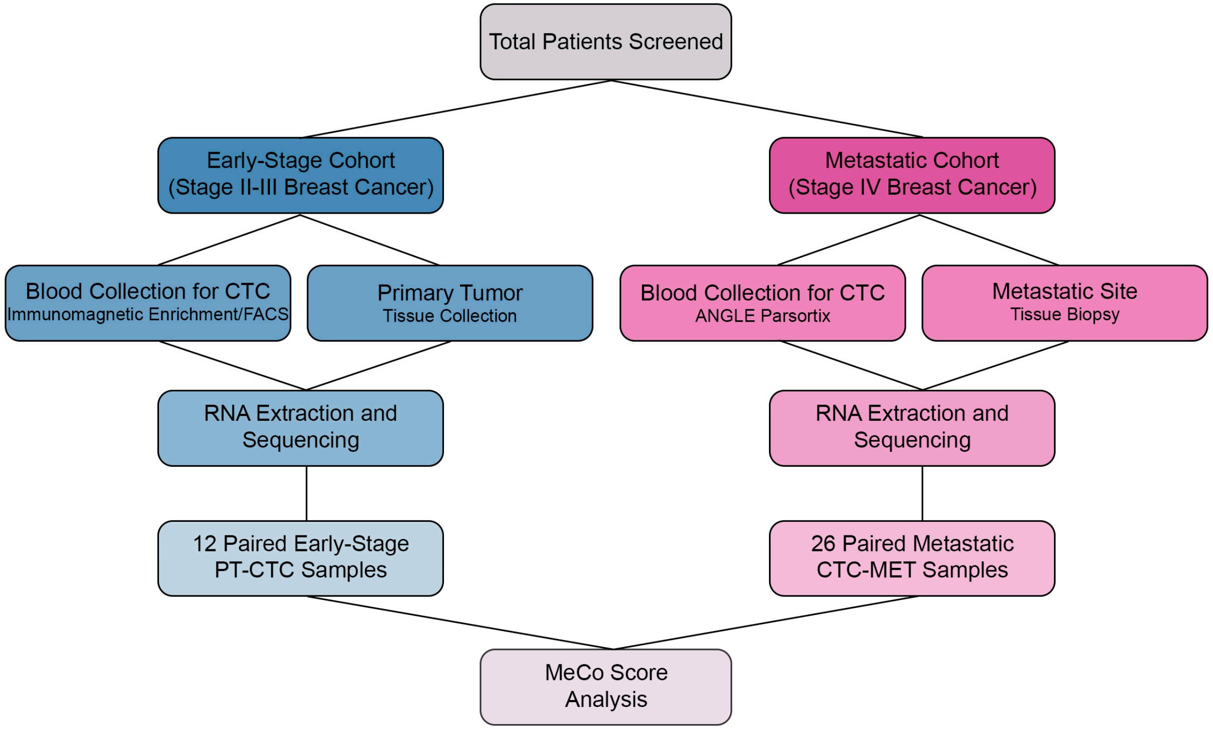

Supplementary Figure S1. Patient Cohort Flowchart. Schematic representation of sample collection and processing workflow for the two patient cohorts in this study. The early-stage cohort (left, blue) consisted of 12 paired primary tumor (PT) and circulating tumor cell (CTC) samples from stage II-III breast cancer patients. CTCs were isolated using immunomagnetic enrichment followed by FACS. The metastatic cohort (right, pink) consisted of 26 paired CTC and metastatic site (MET) samples from stage IV breast cancer patients. CTCs in this cohort were isolated using the ANGLE Parsortix microfluidics system. All samples underwent RNA extraction and sequencing, followed by MeCo score analysis to assess mechanical conditioning throughout the metastatic cascade.

Patient ID	MeCo PT	PAM50 PT	MeCo-CTC	PAM50 CTC	ROR
**Patient #1**	-0.2806034	her2	-0.0764193	lumA	High
**Patient #2**	-0.1023887	Basal	-0.074086	her2	High
**Patient #3**	0.01270623	Basal	-0.1450131	lumB	High
**Patient #4**	-0.0023807	Basal	-0.1021706	lumB	High
**Patient #7**	-0.1952065	Basal	0.06870155	her2	High
**Patient #8**	-0.2586589	Basal	0.10374063	Norm	High
**Patient #9**	-0.2738201	lumA	0.10779196	lumB	High
**Patient #10**	0.0243411	lumA	-0.0324869	lumA	Low
**Patient #11**	-0.2590137	lumA	-0.0326578	Basal	Low
**Patient #12**	0.02880924	lumB	0.1024713	Norm	High
**Patient #13**	-0.1618798	lumB	0.05924332	lumA	High
**Patient #15**	-0.1952326	lumA	-0.0112358	Basal	Low

Supplementary Table S1. Raw data table showing the corresponding PAM50 subtypes in CTCs and PTs, and the ROR in a cohort of patients with early-stage breast cancer.

Patient ID	MeCo CTC	PAM50 CTC	MeCo FollowUp	PAM50 FollowUp	MeCo MET1	PAM50 MET1	MeCo MET2	PAM50 MET2
**101738**	-0.1465914	Her2	-0.168132	LumA	0.45487619	LumB	0.02210341	LumA
**112165**	-0.151753	Her2	0.03062381	LumA				
**112370**	-0.1314125	Basal	0.21968289	LumB	0.11090126	LumA		
**113059**	-0.0064248	Normal	0.10514186	LumB				
**113166**	-0.226953	Basal	-0.0827504	LumA				
**113457**	0.04155804	LumB	0.29822936	LumB				
**113488**	-0.0618689	Normal	0.26606701	LumB				
**19065**	-0.048601	LumA	-0.0457135	Basal	-0.1368012	Normal	0.04501254	LumB
**28089**	-0.0627271	Normal	-0.094283	LumB				
**36541**	-0.1213713	LumA	-0.0648997	Normal	-0.0459298	Normal		
**36978**	-0.1946232	LumA	-0.1813387	Normal	-0.0833546	LumB	-0.1162204	LumB
**68185**	-0.1378423	LumA	-0.2892179	LumB				
**78536**	0.08718221	Her2	0.04762653	LumB				
**78938**	0.07776383	Her2	0.18460296	LumB				
**79388**	-0.0597062	Normal	-0.1123715	Normal				
**79412**	0.00758315	Normal	-0.1088746	Basal	0.17323957	LumB		
**79555**	0.01291655	Normal	-0.030007	LumB				
**79556**	-0.1720988	LumB	0.01002805	LumB				
**79864**	-0.0175712	Basal	0.01878895	Her2				
**80541**	0.01757019	LumB	-0.1594383	LumB				
**81103**	-0.0781181	Her2	0.06728776	LumB				

Supplementary Table S2. Raw data table showing the corresponding PAM50 subtypes in a cohort of patients with metastatic breast cancer. A number of patients had MeCo scores and PAM50 subtype assessed at follow-up and from second metastases.
